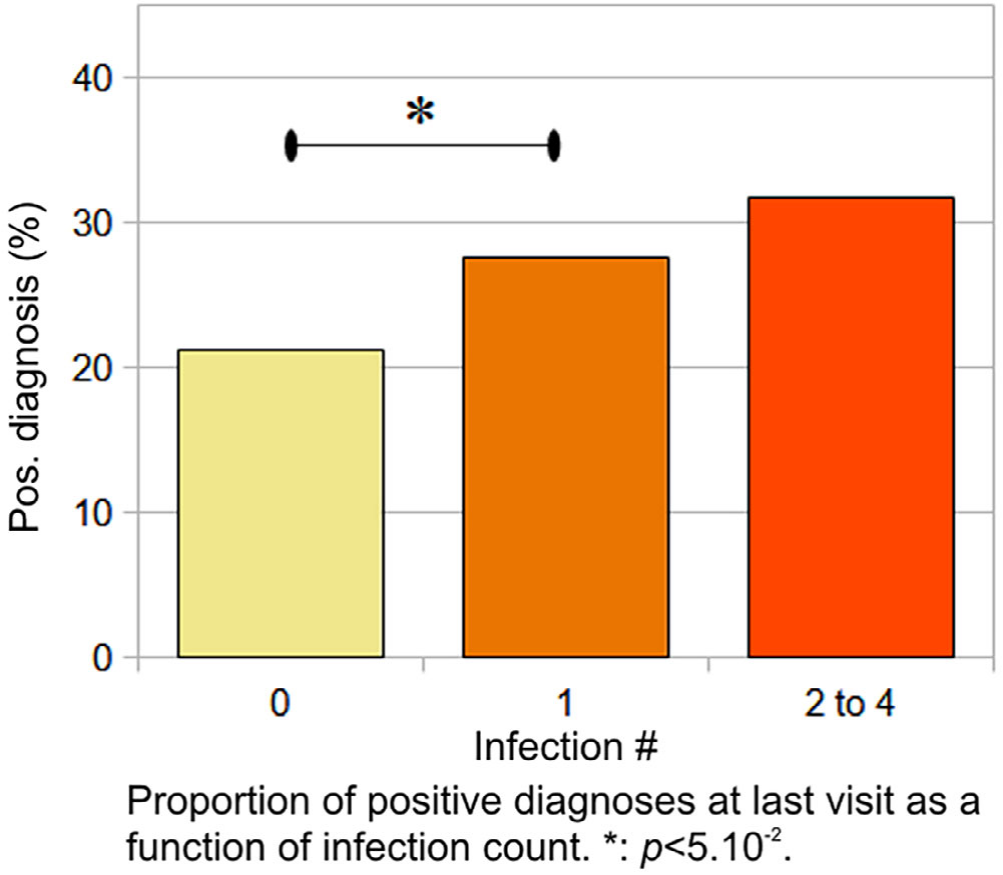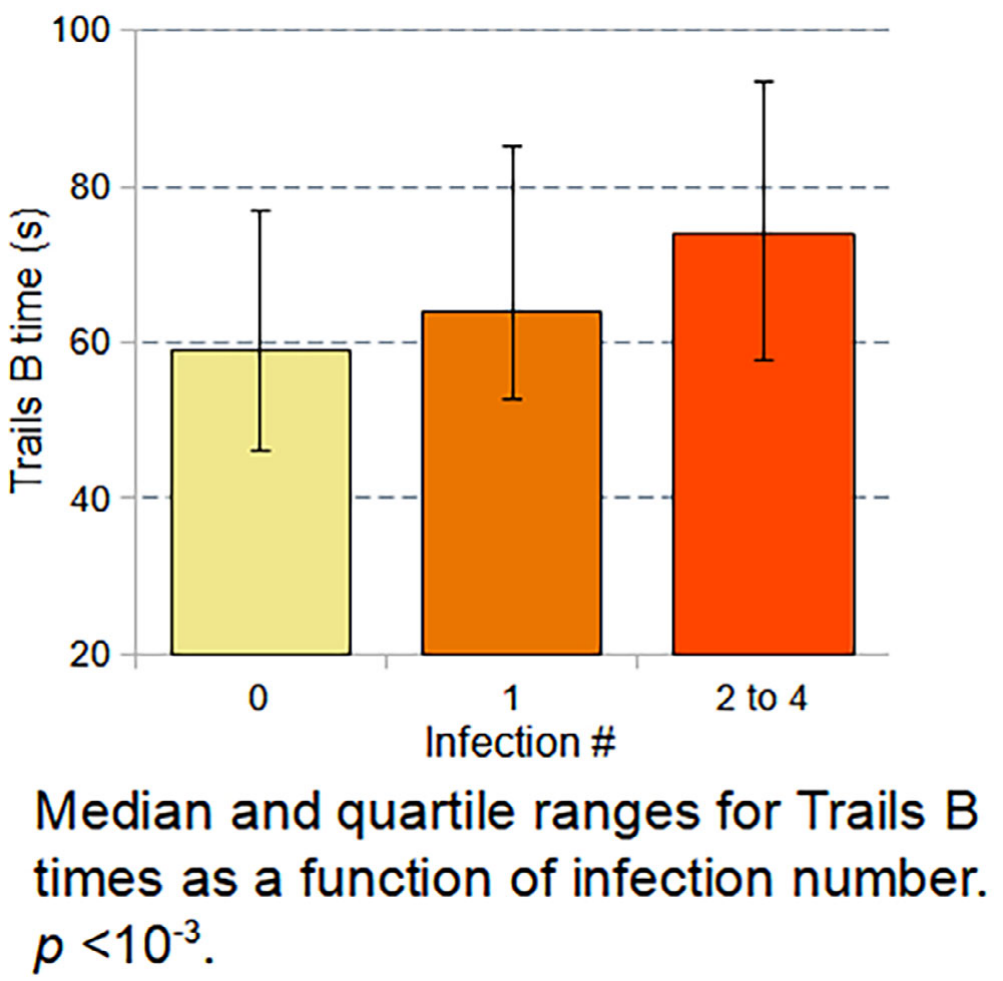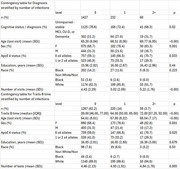# Associations between self‐reported history of infections and cognitive performance: results from the Wisconsin Registry for Alzheimer's Prevention

**DOI:** 10.1002/alz70860_104639

**Published:** 2025-12-23

**Authors:** Patrick S Slama, Adam R Macbale, Erin M. Jonaitis, Rebecca E. Langhough, Sterling C Johnson, Bruno Michel Jedynak

**Affiliations:** ^1^ Freelance, Paris, Ile‐de‐France, France; ^2^ Department of Mathematics and Statistics, Portland State University, Portland, OR, USA; ^3^ University of Wisconsin‐Madison School of Medicine and Public Health, Madison, WI, USA; ^4^ Wisconsin Alzheimer's Institute, University of Wisconsin School of Medicine and Public Health, Madison, WI, USA; ^5^ Wisconsin Alzheimer's Disease Research Center, University of Wisconsin School of Medicine and Public Health, Madison, WI, USA; ^6^ University of Wisconsin‐Madison, Madison, WI, USA

## Abstract

**Background:**

A possible influence of a history of viral and microbial infections on the risk of developing Alzheimer's or other neurodegenerative diseases has been described in the literature (Bu et al., Eur. J. Neurol., 2014; Levine et al., Neuron, 2023). Recent results from the Baltimore Epidemiologic Catchment Area study highlighted the role of viral infections on cognitive performances (Wennberg et al., Alzheimer's & Dementia, 2023). We used the Wisconsin Registry for Alzheimer's Prevention (WRAP, May 2023 freeze) cohort to simultaneously study these two relations based on the infectious burden of participants and their cognitive test performances.

**Method:**

We performed a word search for a history of infections and diseases in the medical history spreadsheets of the WRAP cohort, including viral and bacterial infections and auto‐immune conditions, using medical expertise. Statistical analyses were then performed to compare diagnosis status and cognitive tests at the last visit across infection counts (categorized as 0, 1, or 2+), using the Wilcoxon rank‐sum test, Chi‐square test, and regression analysis over more than 1500 participants. We defined “positive diagnosis” as being clinically classified with dementia, clinical mild cognitive impairment (MCI), or unimpaired‐declining (i.e., “subclinical impairment”) at the last visit. We excluded participants with the impaired but not MCI diagnosis.

**Result:**

Significant differences were observed when comparing the proportions of participants with a positive diagnosis across the various infection counts. The proportions for 0, 1, and 2+ infections were 21.2.%, 27.6%, and 31.7%, respectively (*p* = 0.02). A significant difference was found in the median time for performing the Trails B test, with values of 59 and 72 seconds for participants with no infections and at least two infections, respectively. Regression analysis confirmed the significance of both results, controlling for age, sex, APOE status, number of education years, race, number of visits, and number of tests.

**Conclusion:**

Our analysis shows significant associations between participants’ number of reported infections, their cognitive status, and Trails B results within the WRAP cohort.